# Emerging Roles of Inhibitor of Differentiation-1 in Alzheimer’s Disease: Cell Cycle Reentry and Beyond

**DOI:** 10.3390/cells9071746

**Published:** 2020-07-21

**Authors:** Shang-Der Chen, Jenq-Lin Yang, Yi-Chun Lin, A-Ching Chao, Ding-I Yang

**Affiliations:** 1 Department of Neurology, Kaohsiung Chang Gung Memorial Hospital, Kaohsiung City 833401, Taiwan; chensd@adm.cgmh.org.tw; 2 Institute for Translation Research in Biomedicine, Kaohsiung Chang Gung Memorial Hospital, Kaohsiung City 833401, Taiwan; jyang@adm.cgmh.org.tw; 3 Department of Neurology, Taipei City Hospital, Taipei City 106243, Taiwan; DAB16@tpech.gov.tw; 4 Department of Neurology, College of Medicine, Kaohsiung Medical University, Kaohsiung City 807378, Taiwan; achch@cc.kmu.edu.tw; 5 Department of Neurology, Kaohsiung Medical University Hospital, Kaohsiung City 807377, Taiwan; 6 Institute of Brain Science, National Yang-Ming University, Taipei City 112304, Taiwan; 7 Brain Research Center, National Yang-Ming University, Taipei City 112304, Taiwan

**Keywords:** inhibitor of DNA-binding/differentiation proteins, Alzheimer’s disease, neurodegenerative diseases, cell cycle reentry

## Abstract

Inhibitor of DNA-binding/differentiation (Id) proteins, a family of helix-loop-helix (HLH) proteins that includes four members of Id1 to Id4 in mammalian cells, are critical for regulating cell growth, differentiation, senescence, cell cycle progression, and increasing angiogenesis and vasculogenesis, as well as accelerating the ability of cell migration. Alzheimer’s disease (AD), the most common neurodegenerative disease in the adult population, manifests the signs of cognitive decline, behavioral changes, and functional impairment. The underlying mechanisms for AD are not well-clarified yet, but the aggregation of amyloid-beta peptides (Aβs), the major components in the senile plaques observed in AD brains, contributes significantly to the disease progression. Emerging evidence reveals that aberrant cell cycle reentry may play a central role in Aβ-induced neuronal demise. Recently, we have shown that several signaling mediators, including Id1, hypoxia-inducible factor-1 (HIF-1), cyclin-dependent kinases-5 (CDK5), and sonic hedgehog (Shh), may contribute to Aβ-induced cell cycle reentry in postmitotic neurons; furthermore, Id1 and CDK5/p25 mutually antagonize the expression/activity of each other. Therefore, Id proteins may potentially have clinical applications in AD. In this review article, we introduce the underlying mechanisms for cell cycle dysregulation in AD and present some examples, including our own studies, to show different aspects of Id1 in terms of cell cycle reentry and other signaling that may be crucial to alter the neuronal fates in this devastating neurodegenerative disease. A thorough understanding of the underlying mechanisms may provide a rationale to make an earlier intervention before the occurrence of cell cycle reentry and subsequent apoptosis in the fully differentiated neurons during the progression of AD or other neurodegenerative diseases.

## 1. Introduction

Alzheimer’s disease (AD), a chronic neurodegenerative disorder, is the most common cause of dementia in the aging population. AD is characterized by synaptic dysfunction and ultimate neuronal loss in the cerebral cortex and some subcortical areas, including the hippocampus. The two major pathological hallmarks of AD are the accumulation of extracellular senile plaques and intracellular neurofibrillary tangles with an eventual loss of neurons. The senile plaques are mainly composed of aggregated amyloid-beta peptide (Aβ), the neurotoxic peptide fragment of 39–43 amino acids generated from sequential cleavage of the amyloid precursor protein (APP) by β- and γ-secretase [1,2,3]. Apart from causing neuronal loss, Aβ aggregation also activates several transcription factors, such as hypoxia-inducible factor-1 (HIF-1) [4] and nuclear factor-kappa B (NF-κB) [5], to trigger expression of their downstream genes in neurons. These gene products may either play a detrimental role contributing to Aβ neurotoxicity [6] or, alternatively, represent an endogenous protective response to counteract the harmful effects from Aβ exposure [7]. Understanding these signaling pathways may help to reveal the possible pathogenic mechanisms of AD and facilitate therapeutic advancements.

**Figure 1:**
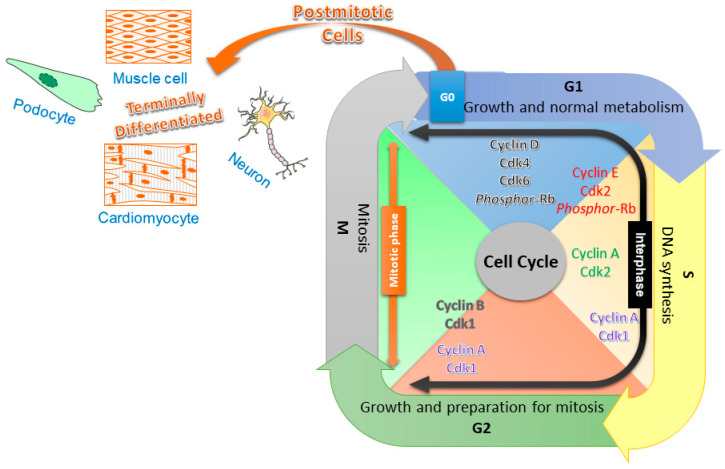
A simplified diagram illustrating the cell cycle progression and its regulators of various phases. The cell cycle is divided into the interphase and mitotic (M) phase; the former is further subdivided into three phases, namely G1, S, and G2. The differentiated cells are without DNA replication and cellular division, remaining in the G0 stage. Upregulated cyclin D binds to and thereby activates Cdk4 and Cdk6 to phosphorylate the retinoblastoma (Rb) protein, allowing cells to escape from the quiescent state and start the G1 phase. The cyclin E-activated Cdk2 phosphorylates an additional Rb protein that promotes the progression of G1 into the S phase. The replication of DNA in the S phase is driven by cyclin A and Cdk2. The cyclin A/Cdk1 complex is found in both the late S and G2 phases, which is suggested to promote chromosome condensation. The formation of the cyclin B and Cdk1 complex regulates the G2/M transition.

The cell cycle is a series of well-defined steps that results in the proliferation of cells (Figure 1). In eukaryotic cells, two major phases of the cell cycle include the interphase and the mitotic (M) phase. During the interphase, which is further subdivided into G1, S, and G2 phases, the cell grows to a larger size to accommodate various newly generated organelles, as well as an additional copy of its genomic DNAs. During the mitotic phase, the genomic DNAs are divided into two sets and separated, along with the cytoplasm, into the two daughter cells [8,9,10]. In the proliferating cells, such as skin, endothelial, and bone marrow cells, the entry of each cell cycle phase is tightly regulated by an exceedingly complex signal transduction network that involves various types of cyclins and cyclin-dependent kinases (CDKs). On the contrary, in most of the nonproliferating cells, including the highly differentiated mature neurons, the cell stays in the quiescent status (or G0 phase), without further progression into the cell cycle. A precisely controlled cell cycle and gene expression are critical for normal and effective neuronal function. The mature neurons must depend on constant surveillance to keep the cell cycle checked in order to maintain the quiescent cellular status [11,12].

Evidence has revealed that neurons at risk of degeneration are also susceptible to restarting a cell cycle process that involves the appearance of cell cycle proteins as well as DNA replication [11,13]. Under pathological conditions such as AD, motor neuron disease (MND), Parkinson’s disease (PD), or brain injury, the interruption of cell cycle checkpoints may result in detrimental consequences. It was observed that neurons returned to the cell cycle process in the early stages of the AD [14]. Several lines of evidence indicate the connection between neuronal loss and cell cycle reentry in AD [15,16,17,18,19]. The underlying mechanism of cycle reentry in mature neuronal cells still awaits further clarification. One of the neurotoxic mechanisms in AD is the accumulation of Aβ that can trigger the occurrence of aberrant cell cycle reentry in the postmitotic neurons, accompanied by the initiation of apoptosis [20].

With an unbiased DNA microarray study, Aβ1-42 has been shown to increase the expression of several genes, including the inhibitor of DNA-binding/differentiation proteins, also known as Id proteins, in neuroblastoma cells [21]. Id proteins, a family of helix-loop-helix (HLH) proteins that includes four members, Id1 to Id4 in mammalian cells, are critical for cell growth and differentiation [22]. Id1 and Id3 appear to be engaged in avoiding uncommitted neural precursor cells to starting a premature process of neuronal lineage commitment and further cellular differentiation. Knockouts of both *Id1* and *Id3* genes reveal premature cell cycle withdrawal and earlier expressions of genes involved in neural determination and differentiation, as well as the increased expression of CDK inhibitors [23]. The expression profiles of *Id2* and *Id4* appear to depend on the timing during development of the nervous system, environmental conditions, and, later, with neural lineage specifications [24,25,26]. The Id proteins do not possess the key motif used for specific DNA binding, as revealed in other basic helix-loop-helix (bHLH) proteins that represent a well-known class of transcription regulators [27,28]. Among them, E proteins were the first identified HLH proteins that bind to the Ephrussi-box (E-box) sequences (5′-CANNTG-3′) [27]. Id proteins often heterodimerize with bHLH proteins, mainly the E proteins, with a dominant-negative action capable of inhibiting DNA binding of these bHLH targets and resulting in transcriptional inactivation [29,30,31].

In addition to the biological activities related to E proteins, Id proteins may also carry functions independent of E protein. In our recent studies, we have shown that Aβ may induce Id1 expression in differentiated rat cortical neurons [32], which contributes to the induction of hypoxia-inducible factor-1 (HIF-1) and the expression of sonic hedgehog (Shh). We further validated that both Id1 and Shh mediate cell cycle reentry and apoptosis induced by Aβ in the fully differentiated postmitotic cortical neurons [33]. Moreover, both Id1 and cyclin-dependent kinase-5 (CDK5) act upstream of HIF-1 to regulate the cell cycle reentry induced by Aβ [34]. In this review article, we address the potential roles of Id1 in AD, which includes cell cycle reentry, apoptosis, and other related mechanisms.

## 2. Id Proteins with Various Pathophysiological Functions

Ever since their cloning three decades ago [28], much of the biological functions of Id proteins has been revealed [35]. Four Id proteins, including Id1 to Id4, exist in mammals, which share a high-sequence homology in the HLH motif and possess one common function, namely inhibiting the DNA-binding activity of E proteins [28,36,37,38]. Outside of the HLH motif, there is very little sequence homology among these four proteins.

Certain bHLH proteins need to compete with Id proteins, forming homo- or heterodimeric complexes with E protein, and bind to the target gene on a particularly recognized motif in the promoter region of the E-box (CANNTG) protein or the N-box (CACNAG) [39], which is critical for the development of specification and differentiation in cells and tissues. In contrast, the inhibitory heterodimer partners, Id proteins, bind bHLH proteins to form a nonfunctional complex, thereby negatively regulating these bHLH factors [22,28]. Genetic and molecular studies in humans and knockout mice reveal that E proteins and Id proteins are of central importance in a wide range of diseases [27].

Transcription factors with a bHLH motif possess the ability to regulate the expression of tissue-specific genes in various organisms. Through the formation of homo- and/or heterodimers, the bHLH proteins exert the function of DNA-binding activity. Proteins encoded by Id-related genes lack the DNA-binding domain. Subsequently, Id proteins carrying the dominant-negative characters heterodimerize with bHLH proteins, mainly E protein, to inhibit binding to DNA and result in transcriptional inactivation [29,30,31]. Id proteins are involved in a number of pathophysiological functions, such as the ability to delay the onset of cellular senescence, to promote the cell cycle, and to increase angiogenesis and vasculogenesis, as well as to expedite cell migration, which is crucial for tumor invasion and metastasis [35]. With the inhibitory effects on cell differentiation, Id proteins also play a crucial role in stem cell maintenance, as well as the formation of innate immune cells [35].

Neural stem cells (NSCs) with a self-renewal ability are competent in generating various cell types in the nervous system [40]. Id proteins are critical for NSC self-renewal by promoting proliferation and preventing differentiation [41], which involve various mediators and mechanisms such as p53 [42] and the bone morphogenetic proteins (BMP)-Smad1 pathway [43], impeding the binding of NeuroD/E47 complexes, and hampering the E-box-mediated gene expression [41].

Several studies showed that various aspects of Id1 are involved in different signaling pathways in the cells of the nervous systems. It was recently demonstrated that, in NSCs, the miR-17-92 cluster is crucial for cognitive function and neurogenesis and may regulate enigma homolog 1/Id1 signaling; ablation of the microRNA-17-92 cluster diminishes the capability of neurogenesis in an adult hippocampus with declined cognitive function, in which Id1 plays a significant role [44]. Cullin-RING E3 ubiquitin ligases, a part of the ubiquitin–proteasome system, are implicated in many cellular processes, such as cell cycle regulation, DNA replication, development, stress responses, and protein quality control [45,46]. Cullin-3, as a component of this system, regulates the Id1 expression that involves intracellular Shh and Wnt signaling in glioblastoma stem cells [47]. In the human neuroblastoma cell line, SK-N-MC, it was found that fibroblast growth factor-2 (FGF-2) can induce Id1 expression at both the mRNA and protein levels; the inhibition of Id1 expression results in an accumulation of FGF-2-treated cells at the G2/M stage and postpones cell death [48]. High expressions of the Id1 protein were found in a rare population of GFAP-positive cells among the subventricular astrocytes in the adult brain with stem cell characteristics, thus defining B1-type adult NSCs [49]. Thrombospondin-1 (TSP-1), as a potent angiostatic factor, regulates crucial angiogenic features of cerebral arteriovenous malformation (AVM). It was reported that TSP-1 is notably downregulated in the cerebral endothelial cells (CECs) of AVM. Adding TSP-1 to AVM-CEC cultures can normalize the abilities of proliferation, migration, and the efficacy of tubule formation. Through studies using siRNA, a mechanistic link between Id1 and TSP-1 was established, in that Id1 negatively regulates TSP-1 expression in AVM-CECs [50]. NeuroD2, a bHLH protein, plays important roles in neuronal development. In the presence of the NeuroD2 protein, Id1 was rapidly and significantly upregulated [51].

Id2 is involved in apoptosis, cell cycle progression, and neural development. Serum and potassium deprivation induce apoptosis in cerebellar granule neurons. The suppression of Id2 expression protects the cerebellar granule neurons from apoptosis, whereas the overexpression of Id2 induces neuronal death [52]. In another study, the overexpression and knockdown of *Id2* respectively increased and attenuated hypoxia/ischemia-induced neuronal apoptosis [53,54]. The effect of Id2 may involve an interaction with tumor suppressor protein Rb, and transcription factor E2F1 and Id2 knockdown could induce G0/G1 cell cycle arrest [54]. In addition to gray matter damage that impairs motor, cognition, and synaptic functions, white matter damage is also critical for neurological recovery after cerebral ischemic stroke. The disruption of white matter integrity, including oligodendrocyte death, myelin loss, and axonal damage, significantly worsens neurological function after ischemic stroke; the impaired proliferation and differentiation of the oligodendrocyte precursor cell may also hamper the functional recovery. Consistently, Id2 is a critical component for regulating the differentiation of oligodendrocyte precursor cells in cerebral ischemia [55].

Similar to Id1 and Id2, Id3 are also expressed in neural cells during development [6], but its roles in the central nervous system (CNS) are less well-studied. Id3 was demonstrated to regulate astrocyte proliferation [56]. In the rats subjected to electrically induced status epilepticus (SE), the expression levels of Id1, Id2, and Id3 proteins in the hippocampus evidently increased in the reactive astrocytes within one day and lasted until three weeks thereafter [57]. Id proteins, mainly Id2 and Id3 but less with Id1, are expressed in astrocyte and microglia that may be involved in modulating cellular responsiveness to tumor necrosis factor-alpha (TNF-α) and CNS inflammation [58]. Id proteins may also be implicated in other physiological functions in the CNS. It was found that the expression of Id1, but not Id2 or Id3, at mRNA and protein levels exhibit changes of several folds during day/night rhythms in the cDNA array analysis of pineal gene expression [59].

Several studies showed the potential roles of Id4 in glioma. It was found that Id4 increases platelet-derived growth factor (PDGF) and nitric oxide synthase 2 (NOS2) expression levels in glioblastoma cells; this positive regulatory circuit of PDGF-NO-Id4 enhances the self-renewal of glioblastoma cells [60]. Id4 also stimulates tumorigenesis in PDGF-induced oligodendroglioma [61] and drives the genesis of glioma-initiating cells via cyclin E and the activation of Notch signaling [62]. Using tissue microarrays and immunohistochemistry, the differential expression of Id4 was found in various grades of astrocytomas, suggesting the possible transformation of low- to high-grade astrocytoma (i.e., glioblastoma) [63]. Id4 was also found to possess proangiogenic function in the growth of glioblastoma [64].

With these abovementioned previous findings of Id family members, together with our own studies demonstrating the critical roles of Id1 in Aβ-induced neuronal cell cycle reentry and subsequent apoptosis, we have made a table (Table 1) to summarize the various biological and pathophysiological functions of these four Id proteins.

## 3. AD and Cell Cycle Dysfunction

The underlying mechanisms responsible for the occurrence of neurodegenerative diseases are not fully delineated. More importantly, at present, there is still no effective treatment for various neurodegenerative disorders, including AD, PD, or Huntington’s disease (HD). Exploring the potential mechanisms relevant to these devastating disorders may help to develop possible therapeutic interventions in the future. Among the possible candidate pathways, cell cycle reentry in AD pathogenesis has attracted the attentions of both clinicians and scientists in basic research.

DNA damage and aberrant activity of the cell cycle in neurons have been detected in various neurodegenerative conditions that may lead to cell demise [65,66]. Several studies showed that marker proteins of the cell cycle progression are expressed in postmitotic AD neurons [15,16,17,18,19]. Cell cycle-related proteins, such as cell division cycle 2 (cdc2), CDK4, cyclin B1, and cyclin D, were observed in AD brains [67,68]. The presenilin-1 (PSEN1) gene encodes the catalytic subunit of γ–secretase. It was reported that the accumulation of cyclin D1 occurs in the familial form of AD brains with PSEN1 mutations, which causes neuronal apoptosis [69]. In neurodegenerative diseases, including AD, some populations of neurons are arrested at the G2/M transition of the cell cycle following the completed DNA synthesis [16]. Despite these observations, precisely how Aβ may be engaged in cell cycle reentry in the postmitotic neurons remains to be fully elucidated. It was assumed that a lack of sufficient ATP formation due to mitochondrial dysfunction is inadequate for further progression into the cell cycles. Thus, neurons may be glued in the middle of the cell cycle at the G2/M checkpoint [70,71] and associated with a greater vulnerability to cell death rather than with finished cell division [11,12]. Mounting evidence reveals that cell cycle dysregulation may represent a prognostic of neuronal apoptosis that may play a significant part in the pathogenesis of AD [67]. It was noted that the appearance of cell cycle-related proteins precedes neuronal cell death in the brains of advanced AD patients, as well as those suffering from mild cognitive impairment (MCI) [14].

## 4. Id1 and AD-A Roles in Cell Cycle Reentry and Cell Death

As Id proteins play a significant role in cell cycle regulation [35], it may be intriguing to understand the potential roles of Id proteins in AD. Recently, we have shown that Id1/HIF-1 and CDK5/HIF-1 contribute to cell cycle reentry in the in vitro AD model of primary cortical neurons [34]. HIF-1, a crucial regulator of mammalian oxygen homeostasis, is composed of an oxygen-sensing HIF-1α subunit and a constitutively expressed HIF-1β subunit [72,73]. The activation of HIF-1 is important for various cellular adaptive responses to tissue hypoxia, such as angiogenesis, cell proliferation, glucose and energy metabolisms, and the synthesis of fatty acids and glycogen, as well as regulation of the pH [74,75]. HIF-1α may also be induced under normoxic conditions in the presence of cobalt chloride, the iron chelator desferrioxamine, or pravastatin in various brain cells [76,77,78]. Aging accompanied with a gradually decreased supply of oxygen and glucose to the brain may be a contributing factor to hypometabolism. Several brain regions critical for cognition and memory preservation, such as the hippocampus, entorhinal cortex, as well as parietal, temporal, and frontal cortices, are more susceptible to such a chronic hypoxic effect with an increased vulnerability [79]. The brain regions with hypometabolism can enhance the expression of APP and lessen the clearance of Aβ, as seen in AD patients [79]. HIF-1α, as a compensatory mechanism, may be induced under such circumstances. Several target genes downstream of HIF-1 are known to enhance cell survival, such as vascular endothelial growth factor (VEGF), erythropoietin (EPO), and inducible nitric oxide synthase (iNOS) [79,80]. However, in our studies, we found that the induction of HIF-1α by Aβ may also lead to cell cycle reactivation, thereby contributing to caspase-dependent apoptosis in the fully differentiated postmitotic neurons [34], suggesting very complicated mechanisms underlying neurodegeneration in AD.

PSEN1 and PSEN2 constitute the catalytic subunits of the γ-secretase complex that is responsible for the cleavage of numerous proteins, including APP, and leads to the formation of the APP intracellular domain (AICD) and Aβ. Aβ aggregation is critically involved in AD pathogenesis [81] and, indeed, Aβ is considered the culprit of AD according to the amyloid hypothesis [82]. It was shown that the level of the HIF-1α protein and its activity of transcription were both diminished in embryonic fibroblasts and in the cortex of forebrain-specific *PSEN1/2* conditional double-knockout mice; the proteolytic γ-secretase function of PSEN1/2 was indeed required for appropriate HIF-1 activation [83]. Thus, PSEN1/2 may also play a direct role in the oxygen-sensing mechanisms via the regulation of HIF-1 in the brain, besides their central roles in the production of Aβ.

Previously, we have shown that Aβ can increase HIF-1α expression [32]. As Aβ can induce Id protein expressions in neuroblastoma cells [21], it is inspiring to investigate the roles of Id protein in primary cortical neurons under Aβ treatment and test whether it is relevant to HIF-1α expression. We have demonstrated that Aβ time-dependently induces Id1, which contributes to HIF-1α stabilization, because the Aβ-mediated induction of HIF-1α can be suppressed by Id1 siRNA. These results denote the regulatory role of Id1 over HIF-1α expression in the context of Aβ-induced neurotoxicity, at least in the cultured rat cortical neurons [32].

Sonic hedgehog (Shh), one of the hedgehog (Hh) family members, was initially identified as a morphogen critically involved in neural development during embryogenesis. In addition to being implicated in neural development, Shh also appears as a vital modulator in adult neural tissues by diverse mechanisms such as anti-inflammation, antioxidation, autophagy, and neurogenesis [84]. Hence, Shh may possibly possess clinical significance in neurodegenerative diseases. Shh functions as a mitogen to adjust the proliferation and facilitate the survival of NSCs/neural progenitor cells (NPCs) [85]. Further, Shh also regulates adult NPC proliferation in the hippocampus, a brain region vulnerable in AD [86,87]. The declined expression of Shh is related with senescence, thus making the brain more vulnerable to aging-related disorders [88]. In our previous study, we have shown that Shh expression was enhanced in the cortex and hippocampus of the aged (12-month-old) brains of APPswe/PSEN1dE9 mice, a commonly used AD transgenic mouse model, as compared to the wild-type littermates; interestingly, the expression of Shh also progressively elevated in the wild-type brains during aging, from 3 to 12 months of age [32]. Further studies in primary cortical neurons validated a signaling pathway of “Aβ → Id1 → HIF-1 → Shh”, at least in vitro.

Aβ can induce aberrant cell cycle reentry in postmitotic neurons with the ensuing induction of apoptosis. Mounting evidence indicates the relation between dysfunctional cell cycle control and neuronal loss in AD. Several marker proteins, including various CDKs and cyclins involved in cell cycle progression, are detected in the postmitotic AD neurons [15,16,17,18,19]. Cell cycle reentry with cyclin D1 accumulation causes cell death in AD brains carrying the *PSEN1* gene mutation [69]. AD neurons with cell cycle reactivation are often arrested at checkpoints before the mitotic phase [16]. Although these observations associate cell cycle dysregulation with neuronal loss in AD, precisely how Aβ may trigger cell cycle reentry in the differentiated postmitotic neurons remains to be fully elucidated. Based on our previous studies concerning Aβ and Id1 [32], we further asked whether Aβ-induced Id1 and Shh expressions contribute to cell cycle reentry and lead to apoptosis in neurons.

In the in vitro AD model, we found that Aβ elicited cell cycle reentry in the postmitotic neurons, as revealed by the expressions of cyclin D1 and phosphorylated retinoblastoma protein (pRb-Pi), two G1-phase markers that include proliferating cell nuclear antigen (PCNA) and the incorporation of 5-bromo-2′-deoxyuridine (BrdU) into newly synthesized DNA, together with histone H3 phosphorylated at Ser-10 as the G2/M marker. Evidently, Aβ also increased the extent of caspase-3 cleavage in the cortical neurons. To further reveal the causal relationship among Id1, Shh, and the cell cycle progression, we used both molecular and pharmacological approaches to validate the Shh/Id1 effects in promoting cell cycle reactivation in the postmitotic neurons exposed to Aβ. We showed that Id1 siRNA, the neutralization antibody against Shh (Shh-Ab), and the CDK-4/6 inhibitor PD0332991 all exerted partial or full competence to attenuate the Aβ-induced cell cycle markers and caspase-3 cleavage in the fully differentiated neurons. Notably, both the recombinant human Id1 protein with an 11-arginine tag on its C-terminal (Id1-Tag), thus rendering it capable of membrane permeation, and the biologically active N-terminal fragment of Shh (Shh-N) were able to increase the expression of cell cycle markers independent of Aβ [33]. These observations further imply that Id1/Shh may represent a common pathway for cell cycle reentry and the accompanied apoptosis in neurodegenerative diseases other than AD. Thus, we have uncovered the vital roles of Id1 and Shh mediating Aβ-dependent cell cycle reentry and, consequently, leading to apoptosis, at least in an in vitro AD model of fully differentiated postmitotic neurons [33].

Given our earlier report demonstrating Aβ-induced Id1 activation with HIF-1α stabilization and subsequent Shh expression in primary rat cortical cultures [32], coupled with the observations that Id1 and Shh both mediate Aβ-dependent cell cycle reentry and apoptosis [33], we therefore were interested to know whether Id1/HIF-1α may also be involved in cell cycle regulation in this experimental paradigm. We found that, in the primary cortical cultures exposed to Aβ, two cell cycle markers, namely cyclin D1 and PCNA, are colocalized with microtubule-associated protein-2 (MAP-2)-positive cells, suggesting cell cycle reentry in the mature neurons [34]. Cobalt chloride, known to stabilize HIF-1α, is sufficient to initiate cell cycle reentry in the postmitotic neurons with the enhanced expression of both cyclin D1 and PCNA independent of Aβ; furthermore, Id1-Tag-mediated cyclin D1 induction can be blocked by HIF-1α siRNA [34]. Overall, these results, along with our earlier studies [32,33], firmly established the signaling cascade of “Aβ → Id1 → HIF-1 → Shh → cell cycle reentry → apoptosis” in the fully differentiated postmitotic neurons.

Cyclin-dependent kinase 5 (CDK5), a proline-directed serine/threonine kinase, is indispensable for proper development of the brain and plays a crucial role in neuronal survival, the phosphorylation of cytoskeletal proteins, and synaptic plasticity [89,90,91]. The activation of CDK5 requires its regulatory subunit and activators, namely p35 and p39 [92,93]. Heightened intracellular concentrations of calcium result in calpain-dependent cleavage of p35 and p39 respectively into the truncated forms of p25 and p29, thereby rendering CDK5 hyperactive with sustained enzymatic activities [94]. CDK5 has been reported to be intimately implicated in the pathogenesis of AD [95]. Aβ may trigger CDK5 activation, leading to hyperphosphorylation of various substrates, including APP, tau, and others [96]. The participation of CDK5 in aberrant cell cycle reentry in neurons has also been described [97,98,99]. Interestingly, CDK5 may physically interact with HIF-1α in neurons, thus contributing to its stabilization [100]. However, whether CDK5-dependent cell cycle reactivation in the postmitotic neurons exposed to Aβ also involves HIF-1α remains unclear. We therefore tested whether CDK5, in addition to Id1 and HIF-1α, may play a role in mediating Aβ-dependent cell cycle reentry in primary cortical neurons. Consistent with the notion of CDK5 acting upstream of HIF-1α, we found that the Aβ-mediated induction of HIF-1α was completely abolished by the CDK5 inhibitor roscovitine, which also suppressed the Aβ induction of cyclin D1 and PCNA, the respective marker for the G1 and S phases during cell cycle progression; further, the siRNA targeting at CDK5 attenuated the Aβ25-35-dependent induction of HIF-1α and cyclin D1. Overall, our results support another signaling cascade of “Aβ → CDK5/p25 → HIF-1α → cell cycle reentry” in differentiated postmitotic cortical neurons [34]. What might be the relationship between these two signaling mechanisms—namely, Id1/HIF-1 and CDK5/HIF-1? Our results indicated that the suppression of Id1 increases the production of p25, while the exogenous application of Id1-Tag decreases the production of p25 in rat cortical cultures. Furthermore, CDK5 suppression by its inhibitor roscovitine and the reduction of p25 expression by the calpain I and II inhibitor MDL28170 both augment Id1 expression in rat cortical cultures [34]. Thus, it appears that Id1 and CDK5 reciprocally suppress the expression/activity of each other in the primary cortical neurons with or without Aβ exposure.

In this review article, we focus on Aβ and its potential link to Id1 to trigger various neurotoxic mechanisms, including cell cycle reentry with resultant neuronal death. While our in vitro evidence using primary cortical neurons points to the neurotoxicity of Aβs as the culprit of AD, this may be somewhat dissonant with a recent article [101], which questions the critical roles of Aβs. Based on neuropsychological testing, serial amyloid PET, and structural MRI examinations, the authors suggest that cognitive alterations may have occurred prior to or during the preclinical stage of amyloid deposition; further, while the brains of MCI patients had higher amounts of Aβs at the onset of the study, this protein did not accumulate faster, as compared to the cognitively healthy individuals. The discrepancies between the in vitro studies and clinical investigations are not clear but may be in part explained by the emerging concept of amyloid-derived diffusible ligands (ADDLs) [102]. According to the amyloid hypothesis [82], AD is characterized by the central deposition of insoluble senile plaques. However, more recent evidence suggests that the plaque alone may be insufficient to fully account for the detrimental actions of elevated Aβs in AD brains. Instead, the soluble oligomers of Aβs, or ADDLs, may play a more predominant role in causing synaptic degeneration accompanied by functional impairments prior to overt neuronal loss. Conceivably, the extent of ADDL accumulations is difficult to be detected by current imaging technologies in clinical settings. In our studies, both aggregated Aβ25-35 and oligomeric Aβ1-42 were used to confirm their effects in triggering neuronal cell cycle reentry and subsequent apoptosis. Nevertheless, we must acknowledge that the mechanisms causing neuronal cell cycle dysfunction are complicated and Aβ must have triggered more complex signal pathways in addition to those identified in our studies, namely Id1, Shh, CDK5/p25, and HIF-1 [32,33,34].

## 5. Conclusion and Future Perspective

**Figure 2:**
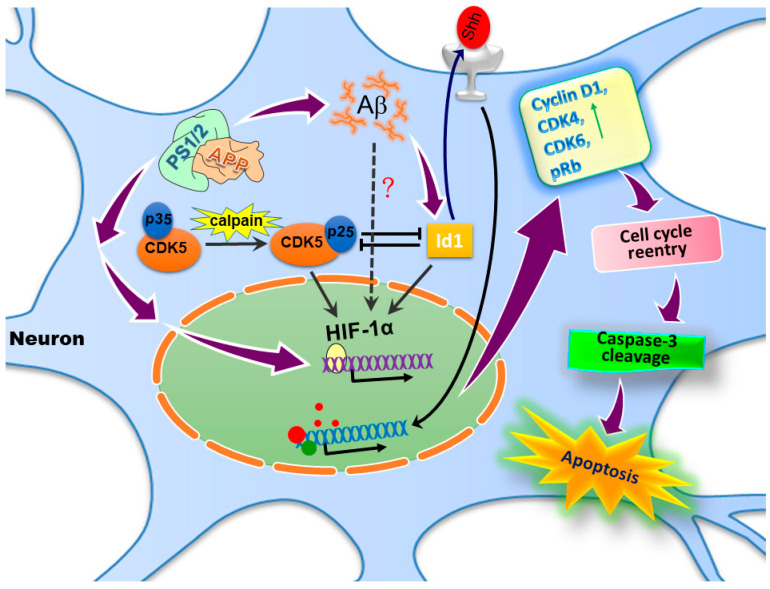
The schematic diagram describes the possible mechanisms of amyloid-beta peptide (Aβ)- and the inhibitor of DNA-binding/differentiation (Id)-1-induced neuronal cell cycle reentry and cell death. The hyperactivity of presenilin (PSEN)-1/2 (PS1/2) and γ-secretase produces abundant Aβs that increase the Id1 expression, which enhances the activation of hypoxia-inducible factor-1α (HIF-1α) and leads ultimately to the expression of the sonic hedgehog (Shh) protein; these mediators together contribute to cell cycle reentry with the expression of cell cycle markers such as cyclin D1 and phosphorylated retinoblastoma protein (pRb) in the postmitotic neurons, which is followed by caspase-3-dependent apoptosis.

AD, the most common neurodegenerative disease in the adult population, leads to progressive memory impairment, gradual mentality decline, behavioral changes, and functional loss due to synaptic dysfunction and neuronal demise in the cerebral cortex and subcortical areas. The underlying mechanisms are complex but may involve both genetic and environmental factors. Id proteins possess many pathophysiological functions, such as regulating cellular senescence, promoting the cell cycle, and increasing angiogenesis and vasculogenesis, as well as accelerating the ability of cell migration, all are critical for tumor invasion and metastasis. However, emerging evidence reveals that aberrant cell cycle reactivation may also play a critical role in Aβ-induced neuronal cell death, which may be a rather earlier event that could offer a rescuing avenue for intervention prior to the apoptotic process. Evidence from our studies revealed the critical effects of Id1 and cell cycle reentry in the in vitro AD model. The proposed potential mechanisms of Id1 in AD are shown in Figure 2. More studies are needed to reveal the possible roles of the Id1 signaling pathway in AD. The potential involvements of Id2, Id3, and Id4 in these pathways are currently still unknown. Is it possible that different members of the Id family play a redundant role in such devastating neurodegenerative diseases such as AD or, alternatively, they possess specific functions distinct from Id1? These issues await further clarification in the future.

## Figures and Tables

**Table 1:** Selected references of Id proteins with various biological and pathophysiological functions in the nervous system.

Inhibitor of DNA-Binding/Differentiation (Id) Proteins	Relevant Biological and Pathophysiological Roles in the Nervous System	Reference and Potential Mechanisms Involving Id Proteins
Id1, Id2, and Id3	Self-renewal and proliferation of cortical neural stem cells	[41]: decrease NeuroD/E47 complexes and E-box-mediated gene expression
Id1	cDNA array analysis of pineal gene expression for circadian rhythm	[59]: Id1, but not Id2 or Id3, mRNA and protein exhibit changes of several folds during day/night rhythms
Id1	Neural stem cell proliferation	[43]: p53 and bone morphogenetic proteins (BMP)-Smad1 pathway
Id1	Involved in neurogenesis and cognitive function	[44]: microRNA-17-92 cluster regulates enigma homolog 1/Id1 signaling
Id1	Involved in intracellular Shh and Wnt signaling in glioblastoma stem cells	[47]: Cullin-3 regulates Id1 expression
Id1	FGF-2 can induce Id1 expression in the human neuroblastoma cell line	[48]: inhibition of Id1 expression results in the accumulation of FGF-2-treated cells at the G2/M stage and postpones cell death
Id1	TSP-1 expression in AVM-CECs	[50]: Id1 negatively regulates TSP-1 expression
Id1	In vivo and in vitro Alzheimer’s disease models	[32,33,34]: Id1, HIF-1, CDK5, and Shh may contribute to Aβ-induced cell cycle reentry in postmitotic neurons; Id1 and CDK5/p25 mutually antagonize the expression/activity of each other (please see Figure 2 for illustration).
Id2	Maintaining normal NPC proliferation	[42]: Id2 functions as a pro-proliferative gene regulated by p53
Id2	Modulation of hypoxia- and ischemia-induced neuronal apoptosis	[53,54]: hypoxia/ischemia upregulates Id2 expression; Id2 knockdown induces G0/G1 cell cycle arrest
Id2	Impaired proliferation and differentiation of oligodendrocyte precursor cells; limited functional recovery after ischemic stroke	[55]: Id2 is a key factor controlling the differentiation of oligodendrocyte precursor cells
Id1, Id2, and Id3	Increased in astrocytes in response to CNS injury	[56]: Id3 was revealed to play a more evident role in regulating astrocyte proliferation in response to injury
Id1, Id2, and Id3	Rats subjected to electrically induced status epilepticus	[57]: expression levels of Id proteins in the hippocampus are increased in the reactive astrocytes
Id1, Id2, and Id3	Modulating cellular responsiveness to TNF-α and CNS inflammation	[58]: putative role for the Id family, expressed in astrocyte and microglia—mainly Id2 and Id3 and less with Id1
Id4	PDGF and NOS2 expression levels in glioblastoma cells	[60,61]: Id4 increases PDGF and NOS2 expression levels; this circuit of PDGF-NO-Id4 enhances the self-renewal of glioblastoma cells and PDGF-induced oligodendroglioma
Id4	Genesis of glioma-initiating cells	[62]: via cyclin E and the activation of Notch signaling
Id4	Was differentially expressed in various grades of astrocytoma	[63]: possible transformation of low-to-high-grade astrocytoma (i.e., glioblastoma)
Id4	The ability of growth of glioblastoma	[64]: Id4 was also found to possess proangiogenic functions

**Abbreviations**: AVM-CECs: arteriovenous malformations-cerebral endothelial cells, BMP: bone morphogenetic proteins, CDK5: cyclin-dependent kinases-5, CNS: central nervous system, FGF-2: fibroblast growth factor-2, HIF-1: hypoxia-inducible factor-1, PDGF: platelet-derived growth factor, NOS2: nitric oxide synthase 2, NPC: neural progenitor cells, Shh: sonic hedgehog, TNF-α: tumor necrosis factor-alpha, Aβ: amyloid-beta peptides, and TSP-1: thrombospondin-1.
